# Basiliximab for early perioperative transplant-associated thrombotic microangiopathy after lung transplantation: a case report

**DOI:** 10.1186/s40792-022-01539-x

**Published:** 2022-09-29

**Authors:** Naohiro Ijiri, Masaaki Sato, Chihiro Konoeda, Kazuhiro Nagayama, Jun Nakajima

**Affiliations:** Department of Thoracic Surgery, Graduate School of Medicine, The University of Tokyo, 7-3-1 Hongo, Bunkyo-Ku, Tokyo, 113-8655 Japan

**Keywords:** Transplant-associated thrombotic microangiopathy, Lung transplantation, Basiliximab

## Abstract

**Background:**

Thrombotic microangiopathy is a syndrome characterized by microangiopathic hemolytic anemia and platelet aggregation, which is caused by endothelial injury, microcirculation thrombosis, and fibrin deposition. Transplant-associated thrombotic microangiopathy rarely occurs after lung transplantation and the onset is generally later than that after bone marrow or other solid organ transplantation. The treatment is to stop administration of the causal agent, which is often a calcineurin inhibitor, such as tacrolimus and cyclosporine. We herein report the case of a patient with early post-transplant thrombotic microangiopathy after lung transplantation treated by introducing basiliximab and temporarily stopping any calcineurin inhibitors until resuming treatment with an alternative calcineurin inhibitor.

**Case presentation:**

A 58-year-old Asian woman underwent bilateral lung transplantation for hypersensitivity pneumonitis caused by an avian antigen, or bird fancier’s lung disease. Postoperatively, she was started on triple immunosuppressive therapy, which included tacrolimus, mycophenolate mofetil, and steroids. On postoperative day 6, she developed thrombocytopenia followed by fever, hemolytic anemia, renal dysfunction, and purpura on her limbs and abdomen. She was diagnosed with transplant-associated thrombotic microangiopathy, and tacrolimus was thought to be the causal agent. We stopped tacrolimus and administered basiliximab. Then, she developed oliguria and needed continuous hemodiafiltration. On postoperative day 14, the platelet count recovered and she was switched from basiliximab to cyclosporine. Using this protocol, worsening thrombotic microangiopathy and acute rejection were avoided.

**Conclusions:**

We report the case of a patient with early post-transplant thrombotic microangiopathy after lung transplantation that was treated with basiliximab. Switching from calcineurin inhibitors using basiliximab may be an option for treating thrombotic microangiopathy without increasing the risk of acute rejection.

## Background

Thrombotic microangiopathy (TMA) is a syndrome characterized by microangiopathic hemolytic anemia (MAHA) and platelet aggregation, and it is caused by endothelial injury, microcirculation thrombosis, and fibrin deposition [1]. Although transplant-associated TMA (TA-TMA) occurs occasionally after bone marrow and solid organ transplantation, it is rare and occurs later after lung transplantation [2]. The most common TA-TMA causal agent is a calcineurin inhibitor (CNI), such as tacrolimus or cyclosporine. Treatment for TA-TMA is to stop administering the causal agent and start an alternative drug, such as another CNI, everolimus, or azathioprine to prevent acute or chronic rejection. Basiliximab is an interleukin-2 receptor antagonist, which is often used as induction therapy in lung transplantation [3]. Basiliximab is also often used to protect kidney function while pausing CNI treatment, which is also called a CNI “holiday,” in patients with acute kidney injury after solid organ transplantations [4]. We herein report the case of a patient with early perioperative TA-TMA after lung transplantation presumably caused by tacrolimus and treated by introducing basiliximab while pausing treatment with all CNIs until treatment with an alternative CNI (cyclosporine) was started.

## Case presentation

**Fig. 1:**
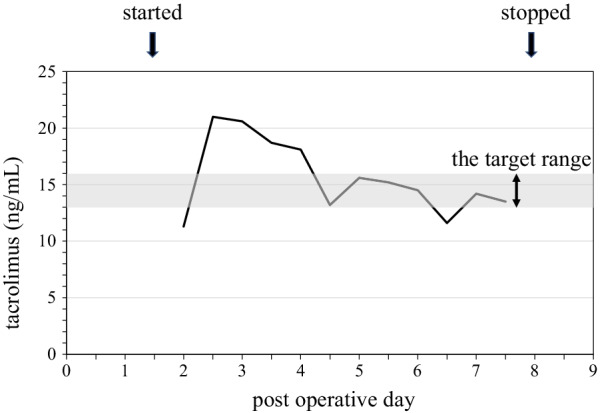
Trends of blood concentration of tacrolimus after lung transplantation. The blood concentration of tacrolimus before presenting TMA was above the target range (13–16 ng/mL) between POD 2–4

**Fig. 2:**
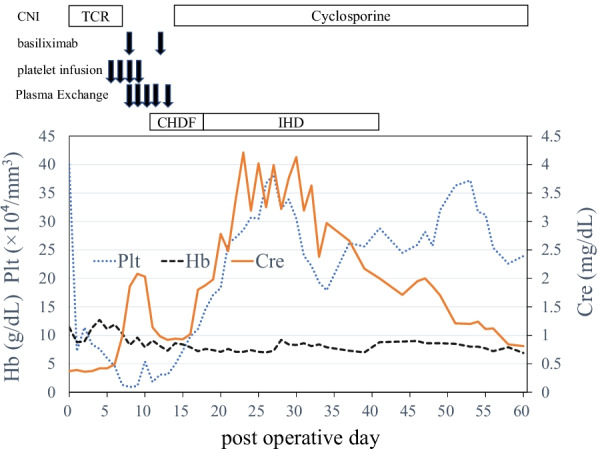
Patient’s clinical course. After stopping tacrolimus and starting basiliximab, the platelet count did not worsen. The patient’s renal function gradually recovered and intermittent hemodialysis was discontinued on POD 41. The creatinine level subsequently decreased to 0.81 mg/dL on POD 60. *TCR* tacrolimus, *CHDF* continuous hemodiafiltration, *IHD* intermittent hemodialysis, *Hb* hemoglobin, *Plt* platelet count, *Cre* creatinine

A 58-year-old Asian woman underwent bilateral lung transplantation for chronic hypersensitivity pneumonitis caused by an avian antigen, or bird fancier’s lung disease. Postoperatively, she was started on maintenance immunosuppressive drugs without induction therapy. The drugs included tacrolimus, mycophenolate mofetil, and steroids. Tacrolimus was initiated in 0.01 mg/kg/day as continuous intravenous infusion and the flow rate was adjusted according to twice-a-day measurements of the blood concentration, the target range of which was between 13 and 16 ng/mL. The dose of mycophenolate mofetil was 1000 mg/day. Regarding steroids, methylprednisolone was started in 250 mg/day from POD1 to POD3 and then the dose was reduced half every 3 days until 62.5 mg/day and then switched to oral prednisolone. On postoperative day (POD) 2, she underwent a redo thoracotomy for intrathoracic bleeding from the chest wall. On POD 6, she developed thrombocytopenia (platelet count decreased from 400,000 to 46,000/mm^3^) followed by fever, hemolytic anemia, renal dysfunction, and purpura on her limbs and abdomen. Laboratory evaluation also showed decreased hemoglobin from 11.4 to 7.9 g/dL, elevated lactate dehydrogenase (LDH) up to 7577 U/L, and increased total bilirubin up to 5.5 mg/dL. Blood smear showed schistocytes, and the haptoglobin level was low (3 mg/dL). On POD 7, the patient’s creatinine level increased to 0.98 mg/dL from 0.37 mg/dL, which was her baseline. On POD 8, the platelet count decreased to 9000/mm^3^ despite daily platelet transfusions. Prothrombin time and activated partial thromboplastin time remained normal. Platelet-associated IgG and heparin-induced thrombocytopenia antibody test results were negative. She was diagnosed with TA-TMA, and tacrolimus was suspected to be the causal agent. The level of blood concentration of tacrolimus was above the target range (13–16 ng/mL) between POD 2–4 (Fig. 1). We then stopped tacrolimus and administered basiliximab starting on POD 8. Plasma exchange was started and repeated five times until the ADAMTS13 (an abbreviation for “a disintegrin and metalloproteinase with a thrombospondin type 1 motif, member 13”, which is a protease that cleaves von Willebrand factor) was shown to be normal. On POD 10, the creatinine level had increased to 2.03 mg/dL and she developed oliguria, and continuous hemodiafiltration (CHDF) was started. On POD 14, the platelet count recovered to 49,000/mm^3^. On POD 15, cyclosporine was resumed, with the target trough levels between 200 and 250 ng/mL. After stopping tacrolimus administration, the platelet count did not decrease further and the patient did not experience acute rejection. On POD 18, CHDF was switched to intermittent hemodialysis, which was eventually discontinued on POD 41. Her renal function recovered gradually, and the creatinine level decreased to 0.81 mg/dL on POD 60 (Fig. 2). On POD 161, she was discharged home; since then, she has not developed another episode of TMA for 3 years.

## Discussion

TMA is a syndrome clinically characterized by MAHA, thrombocytopenia, and organ injury, and it includes many diseases [5]. TMAs are divided into primary TMAs and secondary TMAs. Primary TMAs include thrombotic thrombocytopenic purpura (TTP) and hemolytic uremic syndrome (HUS). Secondary TMAs have a variety of underlying presentations including malignant hypertension, pregnancy, drugs and toxins, viral infection, cancer, autoimmune disease, transplantation, disseminated intravascular coagulation, and metabolic diseases [5].

Hereditary or acquired TTP is usually described as a pentad of fever, neurological and renal impairment, MAHA, and thrombocytopenia. It is caused by a deficiency in the enzyme ADAMTS13, and severely low ADAMTS13 levels (10%) are found in TTP. HUS usually occurs after diarrhea caused by *Escherichia coli* with Shiga toxin [6].

Our patient had worsening anemia (hemoglobin < 9 g/dL or a decrease of ≤ 1 g/dL from baseline), thrombocytopenia (platelet count ≤ 150,000/mm^3^) with schistocytes, and acute renal dysfunction (creatinine ≥ 2 mg/dL or ≥ 120% increase from baseline). The haptoglobin level was abnormally low (< 27 mg/dL), and the serum LDH level was elevated (> 250 IU/L). On the basis of the laboratory data, we diagnosed the patient with TMA in accordance with the criteria indicated in a TA-TMA case series after lung transplantation [1]. Primary TMA was ruled out, because the patient’s ADAMTS13 level was normal and she had no diarrhea. For secondary TMAs, causes other than drugs or transplantation were ruled out, and we finally diagnosed the patient with TA-TMA.

TA-TMA occurs in both hematopoietic stem cell transplant and solid organ transplant recipients. A review showed that the incidence of TA-TMA after lung transplantation was 2.3%, which is less than that of other solid organ transplantations, such as liver or kidney. In addition, TA-TMA following lung transplantation occurred at a median interval of 37 weeks after transplantation, which is later than that for other solid organ transplantations [2]. There are several case reports that described TA-TMA in chronic phases after lung transplantation, and in these cases, patients were treated by switching from tacrolimus to cyclosporine or vice versa, switching from a CNI to everolimus, or discontinuing sirolimus [7–11]. In these cases, the patients did not experience rejection, because the immunosuppressive agents were switched.

Conversely, there are few cases of TA-TMA after lung transplantation in the early perioperative period, such as within 10 days after lung transplantation. One patient, who was diagnosed with TMA related to low ADAMTS13 activity, underwent treatment including changing the CNI from cyclosporine to tacrolimus and administering plasma exchange, but the patient eventually died due to multi-organ dysfunction [12]. Another patient, who was diagnosed with atypical HUS not related to tacrolimus, underwent treatment including induction using everolimus to avoid CNIs and plasma exchanges, but they also died due to sepsis [13].

In our case, discontinuing tacrolimus was the primary treatment choice, because there was a probable cause for TMA. Although directly switching from tacrolimus to cyclosporine was an option, additional renal damage caused by cyclosporine was a major concern, while stopping CNI would increase the risk of acute rejection. In patients with AKI after solid organ transplantation, basiliximab can be used to implement a CNI “holiday” and to prevent acute rejection [4]. Actually, there are two reports regarding basiliximab that was used to reserve CNI after lung transplantation in Japan [14, 15]. One reported the use during treatment for posterior reversible encephalopathy syndrome [14] and the other reported the use as induction therapy for a patient with preoperative renal dysfunction caused by cyclosporine [15]. In liver transplantation, basiliximab is the treatment of choice for TA-TMA to spare CNIs [16]. In our case, we administered basiliximab while pausing CNI administration until recovery from TA-TMA, and we then started cyclosporine. Although cyclosporine also has a similar risk of TA-TMA after lung transplantation [1], switching from one CNI to another seems successful to treat TMA in many cases [2].

Although we used basiliximab to treat TA-TMA, the role of basiliximab in preventing and treating TA-TMA remains unclear. The potential risk of TMA caused by CNI might support routine basiliximab use as an induction therapy, and many lung transplant centers have already implemented this protocol [3]. However, TA-TMA after lung transplantation has been reported even after induction using basiliximab [1]. Considering the potential risks of infection and the issue of health insurance coverage in Japan, we have not used basiliximab as induction therapy in routine treatment after lung transplantation.

In a patient with early perioperative TA-TMA after lung transplantation without induction therapy, basiliximab is a possible therapeutic option to allow pausing CNI to protect renal function and avoid acute rejection.

## Conclusions

We presented the case of a patient with early perioperative TA-TMA after lung transplantation that was treated with basiliximab. Switching from one CNI to another CNI using basiliximab may be an option for treating TA-TMA without increasing the risk of acute rejection.

## Data Availability

The data used in this report are available from the corresponding author on request.

## Abbreviations

TMA: Thrombotic microangiopathy
MAHA: Microangiopathic hemolytic anemia
TA-TMA: Transplant-associated thrombotic microangiopathy
CNI: Calcineurin inhibitor
POD: Postoperative day
LDH: Lactate dehydrogenase
ADAMTS13: A disintegrin and metalloproteinase with a thrombospondin type 1 motif, member 13
CHDF: Continuous hemodiafiltration
TTP: Thrombotic thrombocytopenic purpura
HUS: Hemolytic uremic syndrome
